# Modeling gene regulatory network motifs using statecharts

**DOI:** 10.1186/1471-2105-13-S4-S20

**Published:** 2012-03-28

**Authors:** Fabio Fioravanti, Manuela Helmer-Citterich, Enrico Nardelli

**Affiliations:** 1 Department of Sciences, University of Chieti-Pescara "G. D'Annunzio", Pescara I-65127, Italy; 2 Department of Biology, University of Rome "Tor Vergata", Rome I-00133, Italy; 3 Department of Mathematics, University of Rome "Tor Vergata", Rome I-00133, Italy

## Abstract

**Background:**

Gene regulatory networks are widely used by biologists to describe the interactions among genes, proteins and other components at the intra-cellular level. Recently, a great effort has been devoted to give gene regulatory networks a formal semantics based on existing computational frameworks.

For this purpose, we consider *Statecharts*, which are a modular, hierarchical and executable formal model widely used to represent software systems. We use Statecharts for modeling small and recurring patterns of interactions in gene regulatory networks, called *motifs*.

**Results:**

We present an improved method for modeling gene regulatory network motifs using Statecharts and we describe the successful modeling of several motifs, including those which could not be modeled or whose models could not be distinguished using the method of a previous proposal.

We model motifs in an easy and intuitive way by taking advantage of the visual features of Statecharts. Our modeling approach is able to simulate some interesting temporal properties of gene regulatory network motifs: the delay in the activation and the deactivation of the "output" gene in the coherent type-1 feedforward loop, the pulse in the incoherent type-1 feedforward loop, the bistability nature of double positive and double negative feedback loops, the oscillatory behavior of the negative feedback loop, and the "lock-in" effect of positive autoregulation.

**Conclusions:**

We present a Statecharts-based approach for the modeling of gene regulatory network motifs in biological systems. The basic motifs used to build more complex networks (that is, simple regulation, reciprocal regulation, feedback loop, feedforward loop, and autoregulation) can be faithfully described and their temporal dynamics can be analyzed.

## Background

In order to understand how biological systems behave, a branch of systems biology [1,2] called "executable cell biology" [3] aims to construct computational models which mimic their behavior and which can be used for simulating, in a faithful and cost-effective way, their reactions to external stimuli. The computational model, which is built upon knowledge obtained by performing some *in vitro *experiments, should be complete (it should be able to reproduce all the experimental data) and correct (it should be possible to reproduce its behavior experimentally).

The correspondence between the *in silico *model and *in vitro *observed behaviors is verified by applying model checking techniques [4]. If the model is found to be not consistent with the experimental data, it must be refined and experimentally validated again.

A notable side-effect of the model construction process is that the computational model may suggest new hypotheses about the behavior of the biological system which can then be verified by performing *in vitro *or *in vivo *experiments.

A largely studied class of biological systems is constituted by systems which regulate the expression of genes in an organism. Their behavior is often represented by using gene regulatory networks (GRNs), which describe the interactions among genes, proteins and other components at the intra-cellular level. GRNs have been successful among biologists because they constitute an easy to use and intuitive tool which can be used to represent the biological model under consideration. However, their lack of formal semantics prevents their direct use for performing reliable and consistent simulations and for model checking with experimental data.

There have been several attempts to define formal mathematical and computational frameworks for modeling GRNs. They can be classified into quantitative approaches, using differential equations or stochastic models [5], and qualitative approaches, mostly based on boolean networks [6], Petri nets [7,8], and bayesian networks [9]. See [10] for a detailed analysis and survey of modelling and analysis of GRNs. Motifs have been identified that are significantly overrepresented in biological networks [5,11-14]. The same motifs have been found in organisms at different levels of complexity, ranging from bacteria to humans. The relationships between different types of motifs and their function have been explored in a number of simple cases, *in silico *and *in vivo *[15,16].

Recently, Shin and Nourani [17] have used Statecharts (SCs) [18], a computational framework with a visual language and well-defined semantics, for modeling some small and recurring patterns of interactions in GRNs, called **motifs **[13].

### Gene Regulatory Network motifs

GRN motifs are pattern of interconnections occurring in real GRNs with a frequence that is significantly higher than that in a randomly generated GRN.

Their high frequency suggests that they play an important role in the GRN function and can, thus, be considered as its building blocks.

The functional role of most common GRN motifs has been extensively studied in some organisms, such as *E. coli *and other model organisms [19].

#### The simple regulation motif

The simple regulation motif is one of the most basic interaction patterns. It is composed of two genes *X*, *Y*, where *X *regulates *Y *and the interaction is mediated by a signal *S_X_*. The signal can act as an inducer molecule that binds *X *or can represent a modification of *X *which activates it. Since the regulation of *X *on *Y *is either activation or repression and *S_X _*can mediate the regulation with either presence or absence, four possible types of motifs can be described.

**Figure 1:**
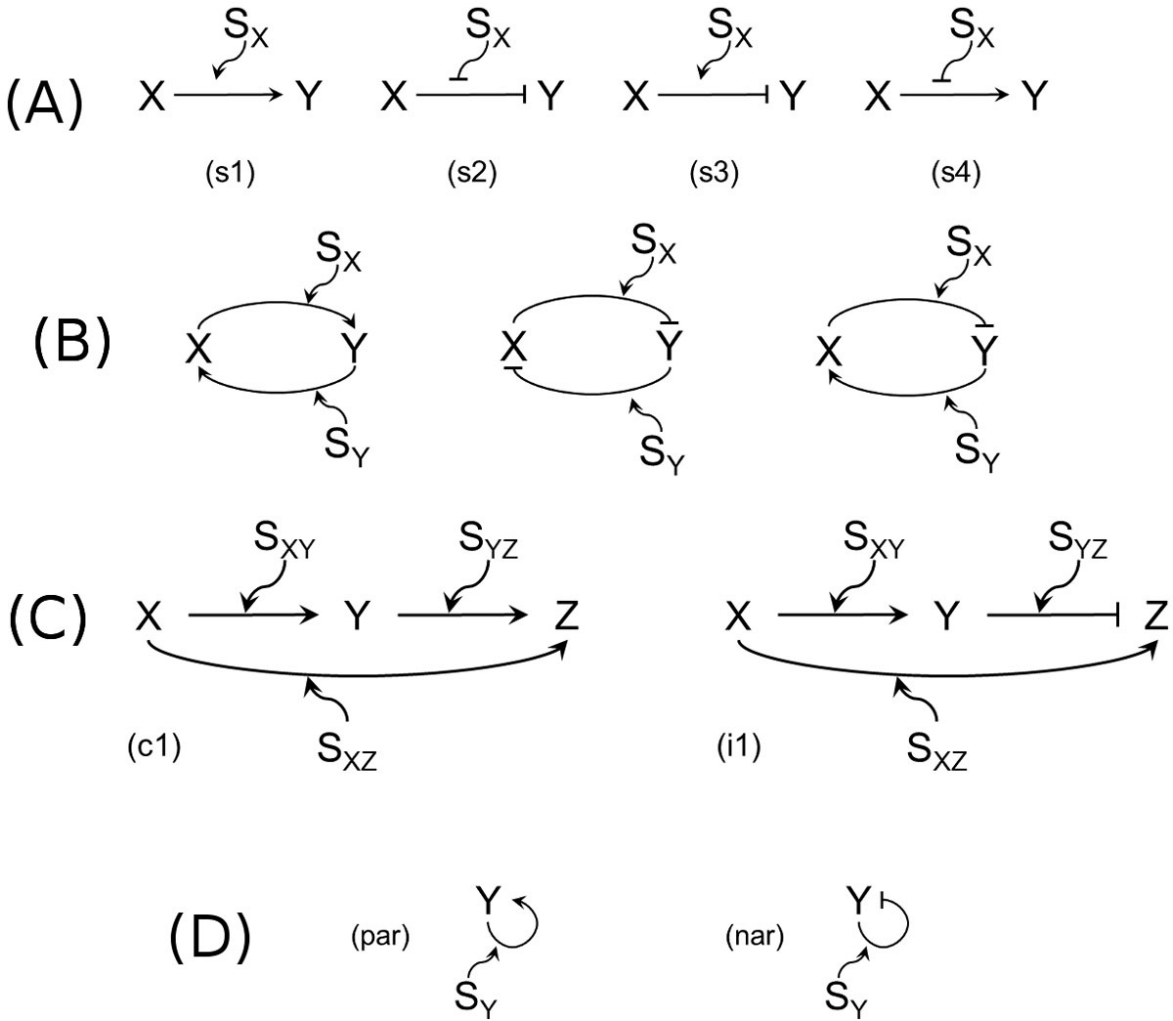
**Regulation motifs**. (A) - The simple regulation motifs: (s1-s2) coherent simple regulation, (s3-s4) incoherent simple regulation. (B) - The feedback loop motifs: double-positive (left), double-negative (middle), and negative (right). (C) - The feedforward loop motifs: (c1) coherent type-1 feedforward loop, (i1) incoherent type-1 feedforward loop. (D) - The autoregulation motifs: (par) positive autoregulation, (nar) negative autoregulation.

A simple regulation motif is **coherent **if both the effects are of the same polarity, i.e. activation of *Y *in presence of *S_X _*(s1 in Figure 1A) or repression of *Y *in absence of *S_X _*(s2). It is **incoherent **if the effects are of different polarity, i.e. repression of *Y *in presence of *S_X _*(s3 in Figure 1A) or activation of *Y *in absence of *S_X _*(s4).

#### The feedback loop motif

The feedback loop motif is composed of two genes *X *and *Y*, which regulate each other, and their interactions are mediated by a signal *S_X _*(for *X *regulating *Y *) and a signal *S_Y _*(for *Y *regulating *X*). Since the reciprocal regulations between *X *and *Y *can be either activations or repressions we have different feedback loop motifs.

A feedback loop motif is **double-positive **if both the reciprocal regulations of the two genes *X *and *Y *are positive, that is, *X *and *Y *activate each other (Figure 1B, left). Similarly, a feedback loop motif is **double-negative **if *X *and *Y *repress each other (Figure 1B, middle). If the effects of the reciprocal regulations of the two genes *X *and *Y *are of different polarity, that is, *X *represses *Y *and *Y *activates *X *or *viceversa*, the feedback loop motif is said to be **negative**. Due to symmetry, we consider only the former negative feedback loop motif (see Figure 1B, right).

#### The feedforward loop motifs

The feedforward loop (FFL) motifs are commonly found in many GRNs of widely studied organisms like yeast and *E. coli*. They are composed of three genes *X*, *Y*, and *Z*, where *X *regulates *Y *and *Z*, and *Y *regulates *Z*. For reasons of simplicity from now on we discuss only the motifs where the regulatory effect depends on the presence of the mediating signals, but our findings apply also to the cases of their absence. Each type of regulation can be either activation or repression. Here we use the term **coherent **(resp. **incoherent**) to denote the case where the sign of the direct regulation from *X *to *Z *is the same (resp. the opposite) as the overall sign of the indirect regulation path through *Y*, as in the seminal paper of Mangan and Alon [20]. Out of the eight possible FFL motifs, the most frequently encountered ones [20] are the coherent type-1 FFL motif c1 and the incoherent type-1 FFL motif i1, both shown in Figure 1C.

The combination of the regulations on gene *Z *by genes *X *and *Y *can be given different interpretations [20]. In the following we will assume that such regulations are combined using the AND logic function, as in the arabinose system of *E. coli *[21]. Although other functions seem to be more appropriate for use in other systems, the AND and OR functions are sufficient to explain the most peculiar properties of FFL.

#### The autoregulation motifs

The characteristic element of an autoregulation motif is a gene regulating itself. The autoregulation motif is **positive **if *Y *activates itself (see par in Figure 1D) and is **negative **if *Y *represses itself (see nar in Figure 1D).

### Statecharts

SCs extend state transition diagrams by adding **concurrency **(i.e., the capability of representing a state as made up by smaller components all active at the same time) and **hierarchy **(i.e., the possibility of representing a state with a set of more detailed substates). The hierarchical structuring capabilities of SCs allow one to model systems at different levels of detail, while concurrency is useful for modeling multiple, mostly independent, portions of a system. Moreover, SCs are **compositional**, that is, they can be defined in terms of other SCs, thus making the specifications more reusable.

These additional features, if correctly exploited, provide a solution to the scalability problems of other computational modeling techniques like, e.g., those based on boolean networks and Petri nets, whose effectiveness rapidly decreases when applied to larger systems [3].

We now summarize some of the SCs features that we believe are essential to understand their potential. Please refer to [18] for more complete and detailed information.

A SC is composed of states and of transitions between states. A state is **composite**, if it contains other states, and is **simple**, otherwise. A composite state is **parallel **if its sub-states are executed concurrently, and is **exclusive **if exactly one of its sub-states is executed. The overall state of a SC is given by all the atomic states currently under execution.

Transitions are used to specify how a system evolves changing its internal state according to the external *stimuli*. They can be labeled by events which trigger their activation and the consequent change of state of the system, conditions for their applicability, and actions to be performed during their execution.

**Figure 2:**
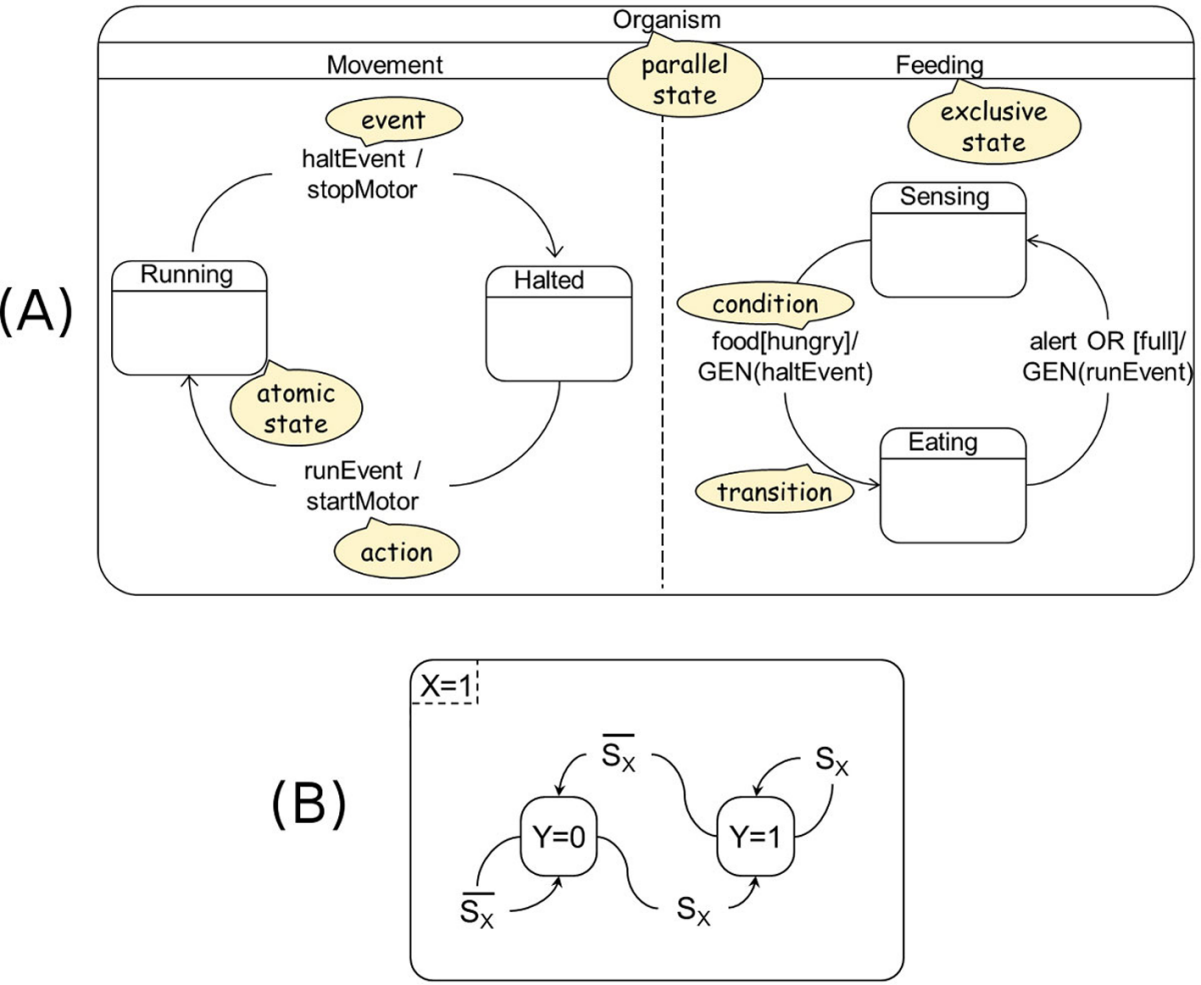
**Statecharts and their use by Shin and Nourani**. (A) - An example of a SC modeling the movement and feeding of an organism: an instance of its overall state is the execution of substates "Running" and "Sensing". (B) - The model according to Shin and Nourani [17] of coherent simple regulations s1 and s2 and their model of autoregulations.

SCs have an intuitive graphical representation: see Figure 2A showing a SC modeling the movement and feeding of an organism by means of two concurrent substates.

SCs have very good software tool support [22-27], which can be used to generate source code (e.g. in Java) whose execution corresponds to the SCs semantics, and to interactively simulate the system execution. SCs have been extensively studied in software and systems engineering, and have demonstrated to be particularly well-suited for modeling and designing reactive systems, that is, systems which evolve reacting to internal or external events, or changed conditions. In the case of GRNs these events can be, for example, the introduction or removal of a protein or of another component.

SCs have also been successfully used to model pancreatic organogenesis in the embryonic mouse [28], cell fate specification during *C. elegans *vulval development [29], and T-cell development in the thymus [30].

Shin and Nourani have used SCs to model GRN motifs [17]. In their approach, each element (gene, protein, signal) can be in one of the two states: "on", which means that the gene is expressed or that the protein is present and active, and "off", which means that the gene is not expressed or that the protein is not present or present in its inactive form.

Moreover, activating interactions in GRNs are translated to transitions from the "off" state to the "on" state for the gene being activated. Similarly, inhibiting interactions correspond to transitions from the "on" state to the "off" state.

Their SCs model of the coherent simple regulation motifs s1 and s2 is shown in Figure 2B, which in their approach represents also the autoregulation motifs.

## Results and discussion

We present an improved method for modeling gene regulatory network motifs by using SCs and we show its application to model a number of motifs. As in the Shin and Nourani [17] approach we use two states "on" and "off" to model each element with the same meaning.

Transitions in our approach are labeled with a logical formula, expressed in terms of presence or absence of genes and signals, which activates the transition when true. Whenever the transitions between "on" and "off" states are not present in our SCs model of a motif this means that the corresponding elements are the independent variables of the modeled motif and their state is possibly changed as a consequence of events outside the motif itself.

A distinctive and novel feature of our method with respect to the method of Shin and Nourani is that we map the elements which are involved in the regulation to concurrent states. This offers a number of advantages that will be detailed in the following.

We also study the temporal behavior of GRN motifs. Given the discrete nature of SCs, the temporal behavior of SCs models of GRN motifs is somewhat rough, but anyhow allows us to simulate some interesting temporal properties of GRN motifs. We are able to model the delay in the activation and the deactivation of the "output" gene in the coherent type-1 feedforward loop motif (c1 FFL), and the pulse in the incoherent type-1 feedforward loop motif (i1 FFL). We are also able to partially model the temporal dynamics of feedback loop motifs and autoregulation motifs, in the sense that the qualitative behavior is represented but the boolean nature of our SCs based approach does not allow us to model more sophisticated temporal mechanisms which require the use of quantitative aspects, like acceleration and damping.

### Model of simple regulation

**Figure 3:**
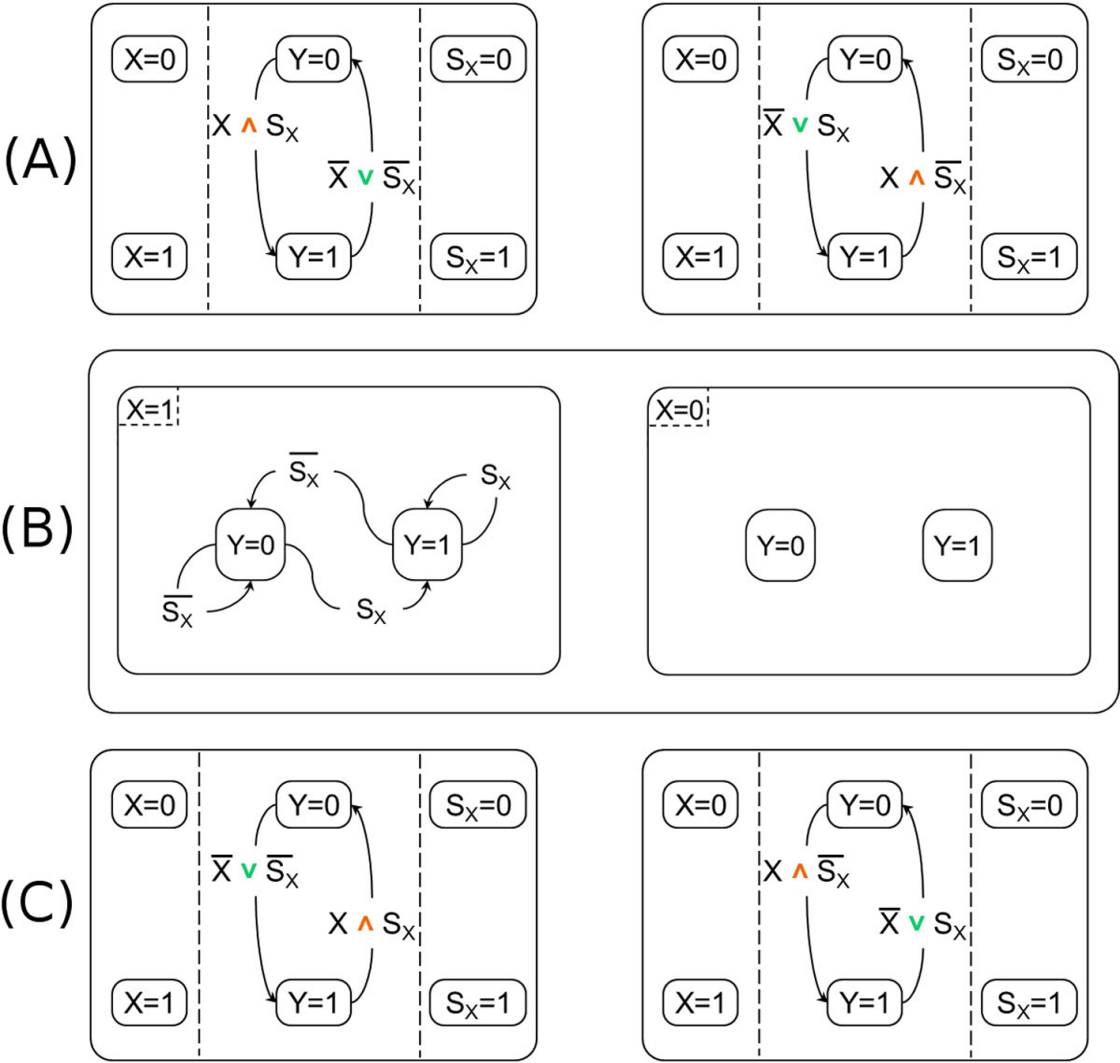
**Our Statecharts models**. (A) - Our models of the coherent simple regulation motifs s1 (left) and s2 (right). The green symbol ∨ represents the logical connective OR and the orange symbol ∧ the logical connective AND. (B) - A possible modification to the Shin and Nourani [17] model of coherent simple regulations trying to solve their incompleteness problem. (C) - Our models of the incoherent simple regulation motifs s3 (left) and s4 (right).

Our models of the simple regulation motifs s1 and s2 are shown in Figure 3A left and right. In both cases, all the elements involved in the regulation, the genes *X *and *Y *and the signal *S_X _*are modeled as concurrent states, and, for each of them, we use two states for modeling its presence (and absence). The activation and deactivation of the regulated gene are modeled by two transitions connecting its presence states, which are triggered according to the truth value of logical formulas depending on the presence of the gene *X *and the signal *S_X_*. Note that in the logical formulas the green symbol ∨ represents the logical connective OR while the orange symbol ∧ the logical connective AND. Note also that in the logical formulas for any element *X*, the expression *X *= 1 is abbreviated as *X *and the expression *X *= 0 is abbreviated as $\bar{X}$.

Our approach for modeling simple regulation is non-ambiguous, because motifs s1 and s2 are represented by two different SCs. See again Figure 3A for our model and compare it with the ambiguity deriving from Shin and Nourani model shown in Figure 2B, where the same SC is used to describe both s1 and s2. Mapping different motifs onto the same SC is a potential source of problems when the mapping is inverted (i.e., from the SC to motifs) because it is not clear whether the SC should be mapped on both the original motifs (thus, possibly leading to an over-specification) or it should be mapped on only one of them.

Moreover the Shin and Nourani model for coherent simple regulations shown in Figure 2B is incomplete, because it implicitly assumes that the regulating gene *X *is always expressed. But ignoring the situation where *X *is not expressed can be significant if, for example, the same gene has a repression role in other parts of the network. If we try to solve their incompleteness problem by adding another state for *X *= 0 then we have to duplicate the states for *Y *= 0 and *Y *= 1, thereby obtaining the SC of Figure 3B and losing the scalability advantage of SCs.

In fact, their model does not fully exploit the concurrency features of SC. This determines sub-optimality, because it does not allow to reduce the size of the system. Their method is therefore not scalable: the complexity of their models grows faster than their size. Moreover, since the states of the regulated gene are modeled as substates of the regulating gene, and not as concurrent states, it is not possible to model networks containing genes which reciprocally regulate each other (see the model of feedback loop presented below). Note that these problems of [17] just described with reference to coherent simple regulations also affect the modeling of the other, more complex, motifs.

Similar considerations also apply to the modeling of the incoherent simple regulation motifs s3 and s4, whose SCs models with our approach are shown in Figure 3C.

### Model of feedback loop

The feedback loop motif is not addressed by the modeling approach defined by Shin and Nourani [17] and we will shortly prove that it cannot be. We first note that the authors themselves observe in the "Further Discussion" section of their paper [17] that feedback loop motif is not part of their modeling scheme and that they intend to incorporate it in the future. We observe that this is not possible in their method, because it requires the states of the regulated gene to be substates of the states of the regulating gene. Since in the feedback loop motif *X *and *Y *act as both regulated and regulating genes, this requirement cannot be fulfilled.

Our modeling approach does not have this limitation because, as already mentioned, the genes and the signals are modeled as concurrent states.

**Figure 4:**
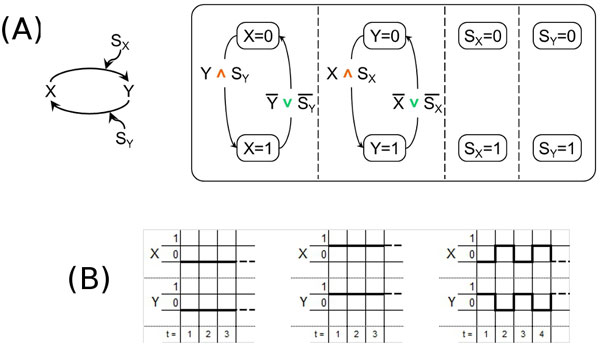
**Double-positive feedback loop motif**. (A) - The double-positive feedback loop motif (left) and its model according to our approach (right). (B) - The temporal behavior of our model of the double-positive feedback loop motif with different initial states for *X *and *Y*.

The double-positive feedback loop motif has two genes *X *and *Y *which reciprocally activate each other. The model for this motif can easily be obtained from the model of the coherent simple regulation motif s1 (previously shown in Figure 3A) by adding the states for the signal *S_Y _*and the transitions between the states for the gene *X *which correspond to the regulation of the gene *X *by *Y*. The resulting model and the motif are shown in Figure 4A.

From now we shall discuss also the temporal behavior of each SCs model representing a given *in vitro *motif so as to determine how closely each model is able to reproduce the corresponding *in vitro *behavior. Note that since a SC is a discrete model the state of the regulated gene at time instant *t *+ 1 depends on the state of its regulating gene at time instant *t*. Also note that the results of this investigation are *a priori *limited by the fact that since our SCs models are boolean any behavior requiring more than two values in the domain cannot be reproduced.

The temporal behavior of the SCs model of the double-positive feedback loop motif is shown in the diagrams reported in Figure 4B. In particular, when *X *and *Y *are initially both present or both absent, it exhibits the "joint bistability" behavior [31], that is *X *and *Y *are either both always "off" or both always "on", as shown in Figure 4B (left and middle). But, as you can see in Figure 4B (right), when the initial state for *X *and *Y *is different, the temporal behavior, due to the approximation of the boolean domain where only two values are available, is not able to escape from the oscillating pattern to fall into one of the two steady states that are known from the *in vitro *experiments [5,31].

**Figure 5:**
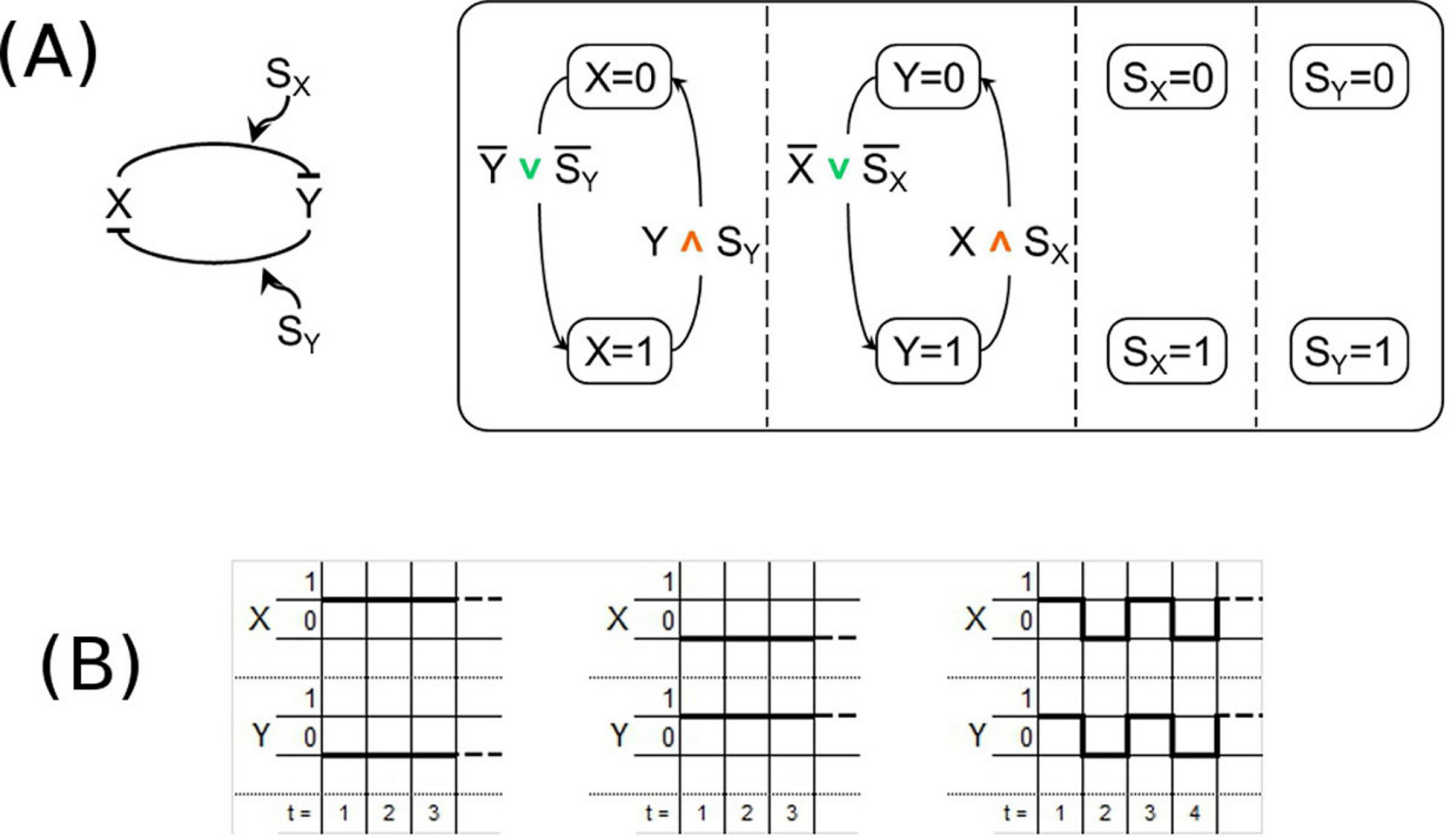
**Double-negative feedback loop motif**. (A) - The double-negative feedback loop motif (left) and its model according to our approach (right). (B) - The temporal behavior of our model of the double-negative feedback loop motif with different initial states for *X *and *Y*.

Our approach allows us also to build the model for the double-negative feedback loop motif, where the two genes *X *and *Y *reciprocally repress each other (see Figure 5A).

Also in this case, our SCs model is able to reproduce the temporal behavior of the motif, that is, *X *always "on" and *Y *always "off", or *viceversa* (this is called "exclusive bistability" in [31]). The corresponding diagrams are reported in Figure 5B (left and middle). Once again, the roughness of the boolean model does not allow the temporal behavior to be attracted into one of the two steady states when the initial states of *X *and *Y *are the same, see Figure 5B (right).

**Figure 6:**
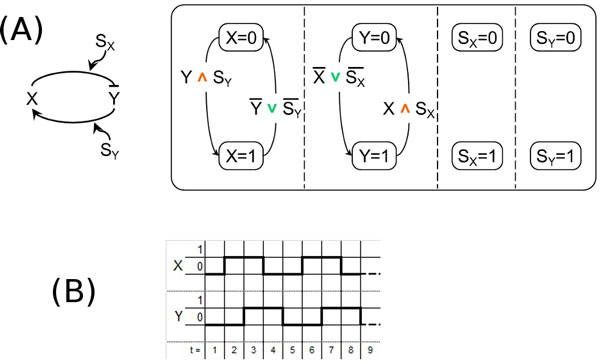
**Negative feedback loop motif**. (A) - The negative feedback loop motif (left) and its model according to our approach (right). (B) - The temporal behavior of our model of the negative feedback loop motif.

For completeness, we also show the SCs model of the negative feedback loop motif (Figure 6A), and the diagram of its temporal behavior (Figure 6B), where the oscillatory behavior known for this kind of motif [32] is reproduced. Some variations of this motif exhibit a damped oscillatory behavior: as said above, the roughness of the boolean model does not allow our modeling approach to reproduce it. We are working on an extension to overcome these limitations.

### Model of coherent feedforward loop

**Figure 7:**
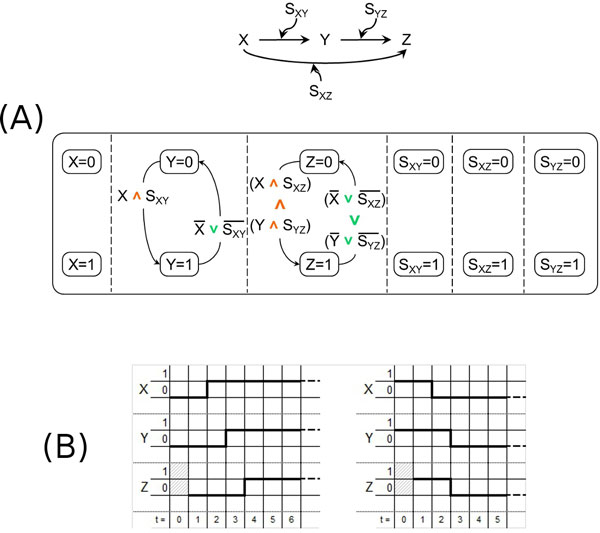
**Coherent type-1 feedforward loop motif**. (A) - The c1 feedforward loop motif (top) and its model according to our approach (bottom). (B) - The temporal behavior of our model of the c1 FFL motif.

The c1 FFL motif with the AND combination of *X*'s and *Y*'s regulations on *Z *has been used as a model of the arabinose system in *E. coli*. This motif, already illustrated in Figure 1C and reported for convenience in Figure 7A (top), can be modeled in our approach by using the SC of Figure 7A (bottom), which, despite its discrete nature, is able to exhibit the same temporal behavior of the *in vitro *system, consisting in (i) a delayed activation of *Z *after the activation of *X*, and (ii) an immediate de-activation of *Z *when *X *disappears (such a behavior is called "sign-sensitive delay" in [13]).

A diagrammatic representation of the temporal behavior of the considered SCs model is reported in Figure 7B, where it can be observed (right) that there is no delay in the deactivation of *Z *(*Z *and *Y *become both inactive at time instant *t *= 3 immediately after *X *disappears at time instant *t *= 2), but its activation (left) is delayed (only *Y *is active in the time instant *t *= 3 right after *X *appears at time instant *t *= 2, and *Z *becomes active only in the step after *Y*'s activation, that is at time instant *t *= 4).

### Model of incoherent feedforward loop

The i1 FFL motif (once again, with the AND combination of *X*'s and *Y*'s regulations on *Z*) has been used as a model of the galactose system in *E. coli *[33] where it produces an impulsive behavior in the regulated gene which first rises very quickly and afterwards soon goes down.

**Figure 8:**
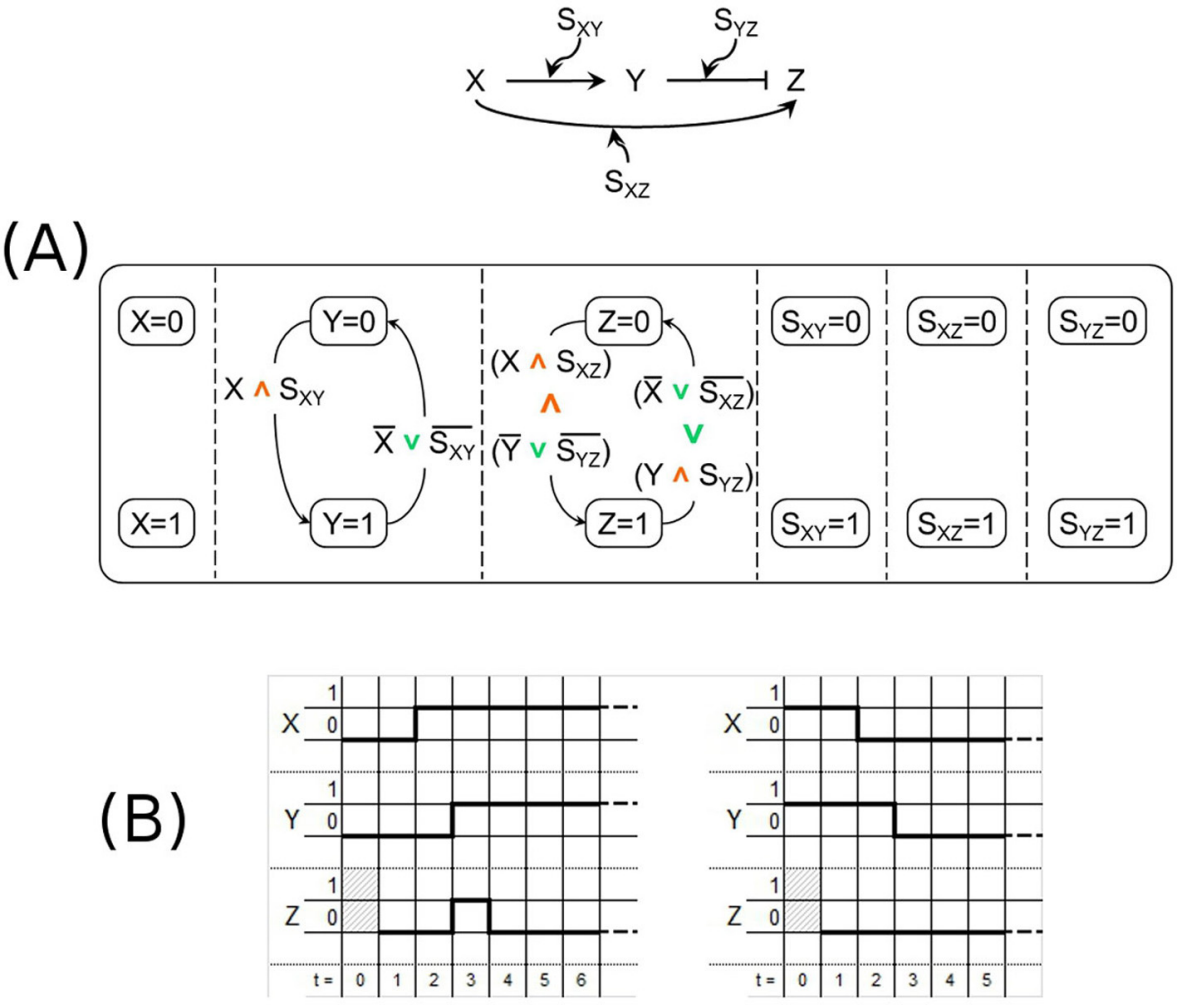
**Incoherent type-1 feedforward loop motif**. (A) - The i1 feedforward loop motif (top) and its model according to our approach (bottom). (B) - The temporal behavior of our model of the i1 FFL motif.

The i1 FFL motif, already illustrated in Figure 1C and reported for convenience in Figure 8A (top), is modeled by using the SC of Figure 8A (bottom) which can reproduce pulse-like dynamics, as shown in the temporal diagram presented in Figure 8B. Soon after *X *becomes active at time instant *t *= 2 (left), also *Z *gets activated at time instant *t *= 3 together with *Y *but, after one more time step, the repressive action of *Y *deactivates *Z *at time instant *t *= 4. Of course, the approximation of the boolean domain only allows a unit time impulse, but that is is enough to show that our SCs model is able to reproduce the dynamic behavior typical of this motif. When *X *becomes inactive at time instant *t *= 2 (right) there is no effect on *Z *which remains inactive, while *Y *becomes inactive in the next step at time instant *t *= 3.

On the other side, our SCs model is not able to express the response acceleration dynamics of the i1 FFL motif with respect to simple regulation [33], as previously said in the discussion of the intrinsic limitation of the boolean domain. We are currently working on the extension of our SCs-based approach to the more general case of a many-valued discrete domain.

### Model of autoregulation

The negative autoregulation motif is a very common and widely studied pattern of regulation.

Experimental results [34] have shown that it behaves as an accelerator of the gene response (with respect to the simple regulation motif), in presence of a high initial concentration of the self-regulating gene. The opposite behavior is exhibited by the positive autoregulation motif which slows down the production of the gene [35].

**Figure 9:**
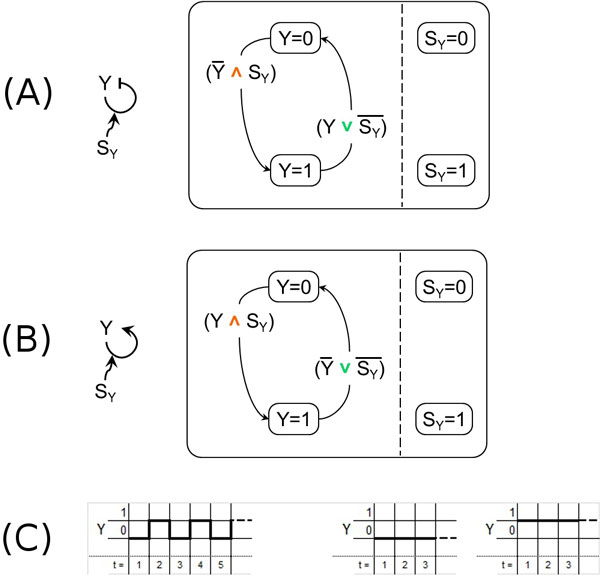
**Autoregulation motifs**. (A) - The negative autoregulation motif (left) and its model according to our approach (right). (B) - The positive autoregulation motif (left) and its model according to our approach (right). (C) - The temporal behavior of our models of the autoregulation motifs: (left) negative autoregulation and (right) positive autoregulation.

Our models for the negative autoregulation motif (see Figure 9A) and the positive autoregulation motif (see Figure 9B) are inherently boolean: therefore they do not have the means of reproducing the acceleration and deceleration which can be observed *in vitro*. The diagrams of their temporal behavior are shown in Figure 9C (left) and (right), respectively. As already mentioned, we plan to extend our modeling approach to take into account these aspects.

**Figure 10:**
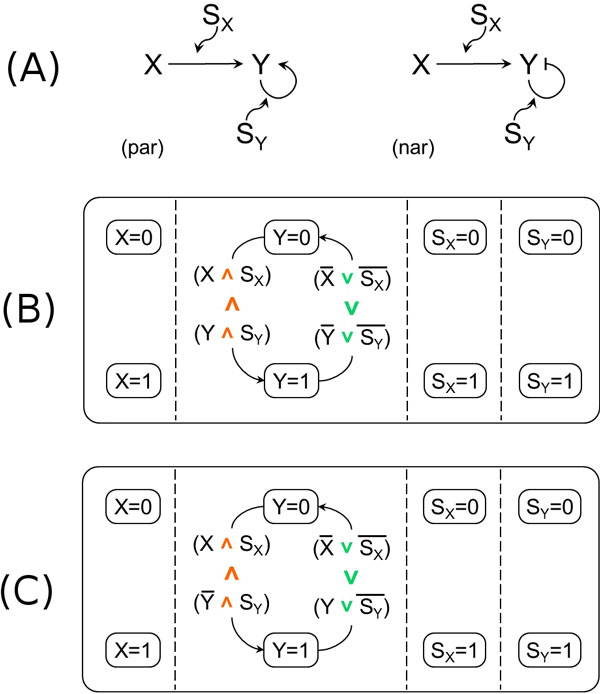
**Autoregulation motifs with an activating gene and our Statecharts model**. (A) - The autoregulation motifs with an additional activating gene *X*: (par) positive autoregulation, (nar) negative autoregulation. (B) - Our model of the positive autoregulation motif with an additional activating gene (shown in (A) at left). (C) - Our model of the negative autoregulation motif with an additional activating gene (shown in (A) at right).

On the other side, note that Shin and Nourani have observed in [17] that with their modeling approach both negative and positive autoregulation are identical to simple regulation in logical domain (see in [17] their Figures 2 and 3 and their discussion of autoregulation). But as you can see by comparing our SCs models for simple regulation (Figures 3A and 3C) to our SCs models for negative and positive autoregulations (to the right in both Figures 9A and 9B), our modeling approach allows to fully distinguish, in the logical domain, the various cases. This is true even if we build with our approach the SCs models for exactly the same autoregulation motifs considered by Shin and Nourani in [17] (shown in Figure 10A) where *Y *is regulated by the AND combination of itself and an additional activating gene *X*. Such SCs models are presented for completeness in Figures 10B (positive autoregulation) and 10C (negative) and the temporal dynamics of *Y *when *X *is expressed is the same shown in Figure 9C.

## Conclusions

We have presented a Statecharts-based approach for modeling motifs of gene regulatory networks which (i) avoids the representation problems (incompleteness, no-concurrency, ambiguity) of a previous proposal [17], (ii) is able to model motifs that were not possible to model by following the approach of [17], (iii) produces more faithful models for the autoregulation motifs than [17], and (iv) is able to exhibit a temporal dynamics which qualitatively follows the actual biological dynamics.

More specifically, we have been able to represent simple regulation, feedforward loop, feedback loop, and autoregulation, which represent the basic motifs that can be used to model more complex networks. Furthermore, our approach, even if intrinsically boolean and discrete, allows us to give a faithful qualitative description of the temporal behavior in the coherent type-1 feedforward loop motif (c1 FFL), in the incoherent type-1 feedforward loop motif (i1 FFL), in feedback loop motifs, and in the positive autoregulation motif.

We are now planning, as future work, to extend our approach to consider also quantitative information, so as to provide a more realistic executable model of GRN motifs and their temporal dynamics.

## List of abbreviations used

GRN: Gene Regulatory Network; SC: Statechart; FFL: FeedForward Loop.

## Competing interests

The authors declare that they have no competing interests.

## Authors' contributions

FF drafted the manuscript and contributed to conceive the work. MHC helped drafting the manuscript and contributed to conceive the work. EN helped drafting the manuscript, contributed to conceive and coordinated the work.
